# Numerical simulation of the transport phenomena in tilted clothing microclimates

**DOI:** 10.1186/2046-7648-4-S1-A68

**Published:** 2015-09-14

**Authors:** Tiago Sotto Mayor, Dinis Oliveira, René Rossi, Simon Annaheim

**Affiliations:** 1 Protection and Physiology Laboratory, Swiss Federal Laboratories for Materials Science and Technology (EMPA), St. Gallen, Switzerland; 2 Bioengineering Department, Engineering Faculty of Porto University (FEUP), Porto, Portugal

## Introduction

Humans depend on clothing protection to minimize the thermal burden imposed on the body by the surrounding environments to which they may be exposed ([1]). The ability of clothing to offer protection depends on multiple factors, from properties of its materials to geometrical aspects influencing the shape of the clothing elements and the way they fit the body. The latter is particularly relevant for the case of loose garments (e.g. CBRN), where relatively thick microclimates exist between the skin and the clothing, which may originate internal buoyancy-driven flows (i.e. natural convection) and substantially alter the way heat is transported to/from the body. Recent literature ([2-4]) report relevant changes in the local heat transport along the skin, in horizontal clothing microclimates, stressing the need for analyses of other geometrical arrangements occurring within clothing.

## Methods

A numerical approach (based on Computational Fluid Dynamics, CFD) was used to study the steady-state transport phenomena within inclined clothing microclimates, formed by air layers trapped between skin and air-impermeable fabrics. Focus was put on the effect of microclimate inclination relative to gravity in order investigate how natural convection affects the local heat transport near the skin, for different body regions (or postures). The analysis addressed both the flow in the microclimate and in the surrounding environment, in order to analyse how heat is transported between the body and the environment.

## Results and discussion

The flow patterns inside clothing microclimates were observed to strongly depend on the inclination of microclimate as it rotates relative to gravity direction. The multi-cell flows observed for the horizontal set-up (i.e. warm skin below the cold fabric), switched to single-cell flows for the vertical set-up, with substantial impact on the temperature maps observed inside the microclimates. This resulted in considerable changes on the local transport rates observed along the skin and along the clothing layers, with clear impact on both the fabric temperature and the external convective coefficient obtained along the clothing surface. The latter was found to differ from the correlation-based convective coefficient curve obtained along surfaces with constant-temperature.

## Conclusion

The obtained results show the importance of the microclimates inclination (i.e. position relative to gravity) in determining the transport processes across clothing. It also stress the importance of considering both the internal flows (i.e. inside clothing) and the external flows (i.e. outside clothing) when analysing or predicting the transport phenomena to/from the body. This is needed to obtain representative insight on the effects influencing the local transport rates between the body and the environment, and is critically relevant when studying ways to optimize the performance of products used near and around the body (e.g. personal protective clothing and equipment, outdoor tents, sleeping bags). For the latter, further investigation is needed to clarify the interactions between the microclimates inclination, dimension and shape relative to the body.
